# Prior activation of social work team process by pre-admission one-click automatic referral system

**DOI:** 10.1093/eurpub/ckac131.105

**Published:** 2022-10-25

**Authors:** S Chon, BE Kim, IJ Lee, GY Choi

**Affiliations:** 1 Department of Emergency Medicine, CHA Bundang Medical Center, Gyeonggi-do, South Korea; 2 Department of Social Work Team, CHA Bundang Medical Center, Gyeonggi-do, South Korea

## Abstract

**Background:**

Coronary artery disease (CAD) is the leading global cause of mortality. Coronary angiography (CAG) is often performed for CAD patients, and the mean length of stay (LoS) is 4 d. Requests to the social work team (SWT) are often delayed until just before discharge, causing unnecessarily prolonged LoS and reduced chances of obtaining financial support. If the SWT’s support system is activated beforehand for scheduled CAGs, the LoS would be shorter and patients would be relieved of emotional and financial burdens.

**Methods:**

Collaborating with the cardiology and medical informatics departments, a one-click automatic referral system (OARS) was set up (January-May 2021). When cardiologists scheduled a CAG in the outpatient department, an OARS cue popped automatically for the medical aid recipients. The cardiologists could also activate the pre-admission OARS for other patients when necessary. Subsequently, the SWT responded on that day to discuss initiating the financial support process with the candidates. The rate of cardiologists’ pre-admission referral to SWT, decision-making time on financial support provision, and proportion of patients receiving financial support were compared before and after initiating OARS.

**Results:**

After initiating pre-admission OARS, the rate of cardiologists’ pre-admission referral to SWT increased from 17.8% (18/101: mean age, 64.6±12.0 y; 32 females; January-December 2020) to 59.1% (55/93: mean age, 64.0±11.8 y; 29 females; June-December 2021) (p < 0.001). Although the decision-making time to provide financial support did not change significantly (8.4±11.1 vs. 4.7±11.6 d; p = 0.96), the proportion of patients receiving financial support increased (45.5% [46/101] vs. 60.2% [56/93]; p = 0.045).

**Conclusions:**

By enhancing the cardiologists’ pre-admission referral to SWT and success rate of receiving financial support, the proactive strategy of pre-admission OARS benefits CAD patients scheduled for CAG.

**Key messages:**

• By building a proactive referral strategy of pre-admission OARS, more CAD patients scheduled for CAG could obtain timely financial support and be relieved both emotionally and financially by the SWT.

• This pre-admission OARS might be incorporated for more disease entities for patients in need.

